# Predictors of ventricular pacing burden after permanent pacemaker implantation following transcatheter aortic valve replacement

**DOI:** 10.1002/clc.23447

**Published:** 2020-09-04

**Authors:** Mohammed Elzeneini, Yazan Assaf, Seyed Hossein Aalaei‐Andabili, Ahmad Mahmoud, Robert Hamburger, Ramil Goel, Anthony A Bavry

**Affiliations:** ^1^ Department of Medicine University of Florida Gainesville Florida USA; ^2^ Division of Cardiovascular Medicine University of Florida Gainesville Florida USA; ^3^ North Florida/South Georgia Veterans Health System Gainesville Florida USA

**Keywords:** pacemaker dependency, pacemaker independency, transcatheter aortic valve replacement

## Abstract

**Background:**

In the era of an expanding use of transcatheter aortic valve replacement (TAVR), conduction disturbances and the requirement for permanent pacemaker (PPM) implantation remains a clinical concern.

**Hypothesis:**

Using a single‐center experience, we sought to identify predictors of ventricular pacing burden after TAVR in patients who required PPM implantation.

**Methods:**

We conducted a retrospective study of 359 consecutive patients with symptomatic severe aortic valve stenosis who underwent TAVR at our institution between September 2013 and July 2019. Thirty patients (8.4%) required a PPM within 30 days after TAVR. Pre and post‐TAVR electrocardiograms, pre‐TAVR echocardiograms and computed tomography (CT), TAVR procedural details and post‐TAVR device interrogation records at 1, 3, and 6 months were reviewed.

**Results:**

Mean percentage of ventricular pacing (VP%) at 1, 3, and 6 months was 58%, 59%, and 56% respectively. Using univariate logistic regression analysis, patients who had low VP% < 5% at 6 months were more likely to have a prosthesis/echocardiography‐derived left ventricular outflow tract (LVOT) diameter ratio < 1.3 (OR 7.00, *P*‐value .048), prosthesis/CT‐derived aortic annulus diameter ratio < 1.02 (OR 7.11, *P*‐value .047), post‐TAVR new‐onset LBBB (OR 16.80, *P*‐value .019), time to PPM implantation greater than 2 days post‐TAVR (OR 9.38, *P*‐value .026) and pre‐TAVR use of a beta blocker (OR 9.40, *P*‐value .026).

**Conclusions:**

In patients who required a PPM implantation post‐TAVR, a lower TAVR prosthesis/LVOT or aortic annulus diameter ratio, post‐TAVR new‐onset LBBB and later time of PPM implantation showed a trend toward predicting a low VP% at 6 months.

## 1 INTRODUCTION

Transcatheter aortic valve replacement (TAVR) is now established as a noninferior alternative to surgical aortic valve (AV) replacement in patients with symptomatic severe AV stenosis, not only in high‐risk^1^, ^2^ and intermediate‐risk patients,^3^, ^4^ but also in patients at low‐surgical risk.^5^, ^6^ Despite the growing evidence of good clinical outcomes with TAVR, conduction disturbances and the requirement for permanent pacemaker (PPM) implantation remains a clinical concern. The incidence rate of PPM implantation within 30 days after TAVR is reported at 6.6% to 8.8% using balloon‐expandable valves in the PARTNER 1, 2, and 3 trials,^3^, ^5^, ^7^ and remains at a high rate of 17.4% using self‐expandable valves in their latest low‐risk trial.^6^

These conduction disturbances are believed to be due to mechanical stress from the prosthetic valve on the conduction system in the atrioventricular node and interventricular conduction system,^8^ and include variable degrees of atrioventricular block, bradycardia and left bundle branch block (LBBB). Recent studies have shown a trend in recovery of some of these conduction disturbances, resulting in a decrease in the percentage of patients who are PPM dependent with time.^9^ Persistence of PPM dependency has been reported to be somewhere between 22% and 64% in recent single‐center studies.^10^, ^11^, ^12^, ^13^, ^14^, ^15^ A better understanding of the natural course of these rhythm disturbances and identifying predictors of their recovery can be particularly useful in helping the clinical decision‐making process when a PPM is being considered for certain disturbances where a strong consensus is not available. Recent institutional experiences have identified factors as a pre‐TAVR right bundle branch block (RBBB),^10^, ^11^, ^14^ higher prosthesis/LVOT diameter ratio,^14^ and earlier time to PPM implantation within 1 to 2 days post‐TAVR^10^, ^13^, ^14^ as predictors of pacemaker dependency. In this study, we aimed to use our institutional experience to identify predictors of post‐TAVR ventricular pacing burden with time, and investigate predictors of a low‐ventricular pacing percent over a 6 month follow‐up period that may suggest pacemaker independency.

## 2 METHODS

### 2.1 Study population

We conducted a single center retrospective cohort study of 359 consecutive patients with symptomatic severe AV stenosis who underwent TAVR at Malcolm Randall VA Medical Center, Gainesville, Florida between September 2013 and July 2019. These patients were treated using either the balloon‐expandable Edwards Sapien (original, XT or 3; Edwards Lifesciences, Irvine, California) or the self‐expanding Medtronic valve (CoreValve, Evolut R or Evolut PRO; Medtronic, Minneapolis, Minnesota) using percutaneous femoral or alternative arterial access. For our study analysis, patients who required a PPM implantation within 30 days after the TAVR procedure were included, while patients who already had a prior PPM were excluded. The final study cohort for analysis included 30 patients. The study protocol was approved by our local Institutional Review Board, and obtaining patient consent was waived.

### 2.2 Data collection

All data was obtained from electronic medical records. Pre‐TAVR echocardiograms were reviewed for left ventricular (LV) function, LV outflow tract (LVOT) diameter and AV area. Computed tomography (CT) TAVR protocol images were reviewed for aortic annulus perimeter‐derived diameter. TAVR procedural details were reviewed for valve prosthesis type and size, use of preimplant valvuloplasty, use of postimplant dilatation and AV hemodynamics. Prosthesis/echocardiography‐derived LVOT diameter ratio and prosthesis/CT‐derived aortic annulus diameter ratio were calculated. A supplementary figure (Supplementary Figure 1) demonstrates how these diameters were obtained. Pre and post‐TAVR electrocardiograms (ECGs) were analyzed for heart rate, rhythm, PR interval, QRS duration, and the presence of any conduction disturbances. Indications for which PPMs where implanted were collected from clinical documentation and ECG interpretation by a cardiology electrophysiologist and categorized as: persistent complete or high‐degree atrioventricular block (AVB), transient complete or high‐degree AVB, pause(s) more than 3 seconds and symptomatic bradycardia (including sick sinus syndrome, atrial fibrillation with slow ventricular rate and prolonging PR interval).

### 2.3 Follow‐up

Patients were retrospectively followed up for a duration of 6 months. Device interrogation records from outpatient visits were reviewed at 1, 3, and 6 months after PPM implantation. All the PPMs were programmed in the DDD mode at 60/min or the VVI mode in cases of persistent atrial fibrillation. The percentage of ventricular pacing (VP%) at each visit was documented, and ventricular pacing burden was categorized as low (VP% less than 5%), intermediate (VP% between 5% and 95%) and high (VP% more than 95%). These cutoffs were previously used and interpreted as VP% less than 5% suggesting pacemaker independency and VP% more than 95% suggesting absolute pacemaker dependency.^10^

### 2.4 Statistical analysis

For descriptive analysis, continuous variables were presented as mean values ± SD, and categorical variables were presented as counts and percentages. Mean values of continuous variables were compared between the three groups of VP% by one‐way analysis of variance (ANOVA) or Kruskal Wallis test, while categorical variables were compared by Fisher's exact test. Univariate logistic regression analysis was performed to test for predictors of low VP% < 5% at 6 months, followed by a multivariable logistic regression model including all significant factors on univariate analysis. Significant continuous predictors were dichotomized using the highest C statistic from a Receiver operating characteristic (ROC) curve to identify clinically useful cutoff values. Odds ratio was generated for all prediction values, and a significance level of *P* < .05 was used to indicate statistical significance. The statistical software SPSS (IBM SPSS Version 26 for Mac) was used for all statistical analysis.

## 3 RESULTS

A total of 30 out of 359 patients who underwent TAVR (8.4%) required a PPM within 30 days, with a median time to PPM implantation of 2 days post‐TAVR (interquartile range 1 to 6 days). All but one patient were males (97%), and the mean age was 76 ± 8 years. The majority of valves were Edwards Sapien (original, XT or 3; 77%). Indications for PPM implantation were persistent high‐degree AVB (n = 13, 43%), transient high‐degree AVB (n = 6, 20%), pause more than 3 s (n = 4, 13%) and symptomatic bradycardia (n = 7, 23%). Mean VP% at 1, 3, and 6 months was 58%, 59%, and 56%, respectively, with no significant change over the 6‐month follow‐up period. None of the patients was lost to follow‐up, but four died within the follow‐up period. The final cohort for comparison of patients according to VP% at 6 months included 26 patients, categorized into low VP% <5% (n = 7), intermediate VP% 5% to 95% (n = 10) and high VP% >95% (n = 9).

**FIGURE 1:**
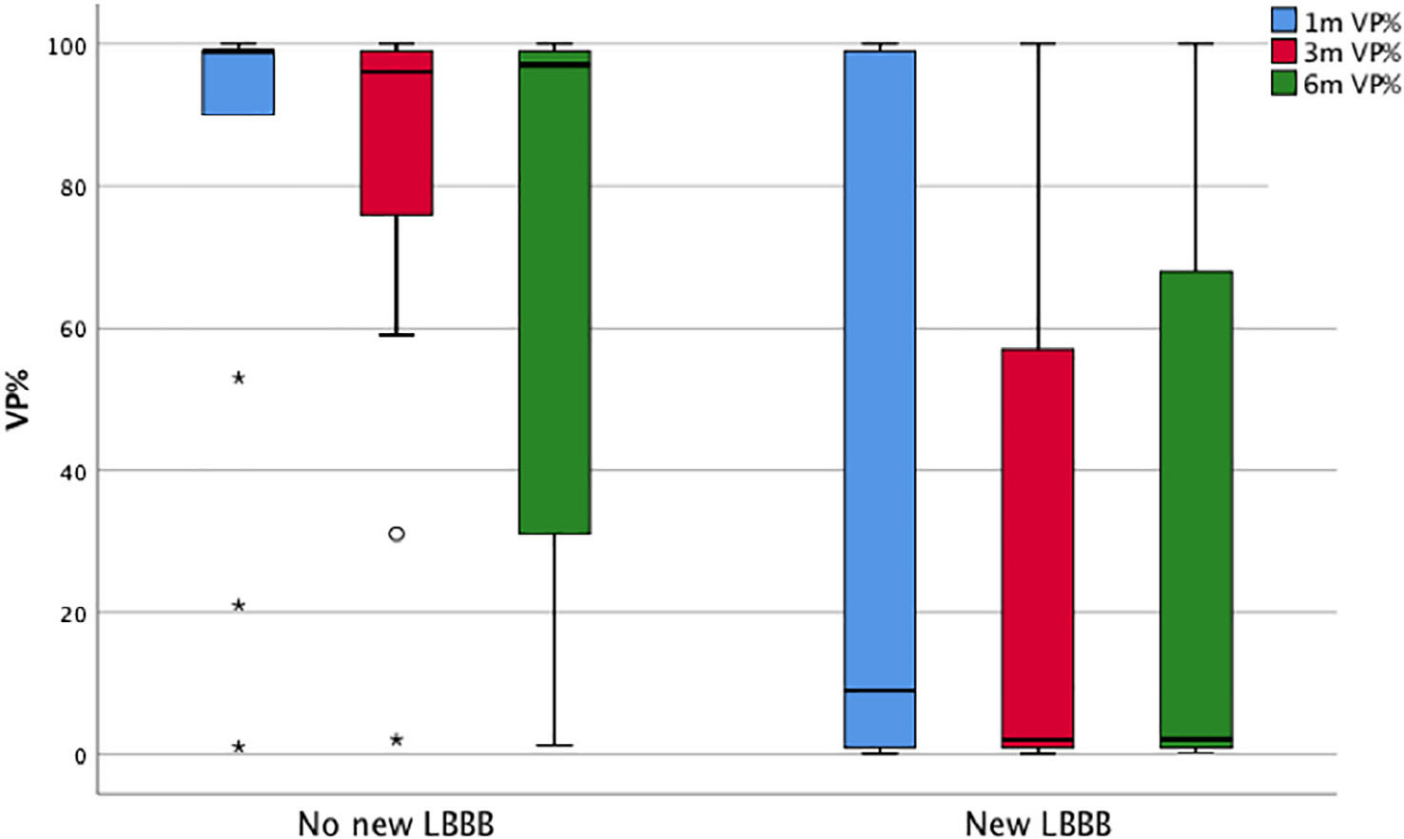
Difference in median (IQR) percent of ventricular pacing at 1,3, and 6 months after TAVR‐related PPM implantation in patients with and without new‐onset LBBB. LBBB, left bundle branch block; PPM, permanent pacemaker; TAVR, transcatheter aortic valve replacement; VP%, ventricular pacing percentage

Table 1 summarizes the preoperative baseline characteristics, medication use, ECG details and echocardiographic findings of patients in the three groups. All variables were tested for differences between the groups, and prosthesis/LVOT diameter ratio was the only variable meeting statistical significance (*P*‐value .021, with mean values of 1.21, 1.37, and 1.39 in the low, intermediate and high‐VP% groups, respectively). Prosthesis/aortic annulus diameter ratio showed a similar trend but the difference between groups did not reach statistical significance (mean values of 1.03, 1.08, and 1.12, respectively). Table 2 summarizes the differences in operative details, valve prosthesis type and size, hemodynamic measurements, postoperative ECG details and PPM implantation time and indication in the three groups. Statistically significant differences in the groups were found with post‐TAVR new‐onset LBBB (*P*‐value .039, being present in 86%, 30%, and 22% in the low, intermediate and high‐VP% groups, respectively), and time to PPM implantation (*P*‐value 0.031, with mean time of 8, 3, and 2 days post‐TAVR in the low, intermediate and high‐VP% groups respectively). Patients with new‐onset LBBB had a lower median VP% at 1, 3, and 6 months compared to those without as shown on a boxplot in Figure 1 (*P*‐value 0.012, 0.012, and 0.054 respectively using Mann‐Whitney *U* test).

**TABLE 1:** Pre‐TAVR baseline clinical, electrophysiological, and anatomical characteristics of patients with low, intermediate and high percent of ventricular pacing at 6 months

	All patients	VP% <5%	VP% 5‐95%	VP% >95%	*P*‐value
	(n = 26)	(n = 7)	(n = 10)	(n = 9)	
Baseline clinical characteristics					
Age, years	76 ± 8	74 ± 5	75 ± 11	80 ± 7	.298
Male gender	25 (96)	7 (100)	10 (100)	8 (89)	.615
Body mass index, kg/m^2^	29.6 ± 6.6	32.5 ± 7.3	29.7 ± 4.3	27.4 ± 7.8	.313
Hypertension	22 (85)	6 (86)	8 (80)	8 (89)	.000
Diabetes	12 (46)	4 (57)	4 (40)	4 (44)	.885
Coronary artery disease	17 (65)	4 (57)	5 (50)	8 (89)	.188
Prior myocardial infarction	3 (12)	2 (29)	1 (10)	0	.245
Prior CABG	6 (23)	3 (43)	1 (10)	2 (22)	.312
Prior PCI	5 (19)	2 (29)	2 (20)	1 (11)	.828
Heart failure	8 (31)	2 (29)	3 (30)	3 (33)	.000
Stroke or TIA	4 (15)	3 (43)	1 (10)	0	.069
Peripheral vascular disease	8 (31)	3 (43)	2 (20)	3 (33)	.666
COPD	7 (27)	3 (43)	3 (30)	1 (11)	.347
Chronic liver disease	1 (4)	1 (14)	0	0	.269
Serum creatinine	1.4 ± 1.4	2.1 ± 2.6	1.0 ± 0.3	1.3 ± 0.3	.227
Beta blocker use	9 (35)	5 (71)	2 (20)	2 (22)	.084
Calcium channel blocker use	7 (27)	2 (29)	3 (30)	2 (22)	.000
Amiodarone use	1 (4)	1 (14)	0	0	.269
Digoxin use	1 (4)	1 (14)	0	0	.269
Pre‐TAVR electrocardiography					
Heart rate, beats/min	70 ± 13	68 ± 12	72 ± 17	69 ± 8	.811
Atrial fibrillation	5 (19)	1 (14)	4 (40)	0	.090
PR interval, ms	220 ± 66	215 ± 50	193 ± 48	244 ± 83	.364
Mobitz type I	1 (4)	0	0	0	.615
QRS durations, ms	119 ± 28	108 ± 12	128 ± 35	119 ± 28	.393
RBBB	12 (46)	1 (14)	6 (60)	5 (56)	.156
RBBB + LAFB	4 (15)	0	2 (20)	2 (22)	.515
RBBB + LPFB	3 (12)	0	2 (20)	1 (11)	.758
Trifasicular block	4 (15)	0	1 (10)	3 (33)	.272
IVCD	2 (8)	2 (29)	0	0	.065
Pre‐TAVR echocardiography					
LVEF, %	58 ± 7	59 ± 3	58 ± 4	57 ± 11	.775
LVESd, mm	32 ± 6	30 ± 6	33 ± 7	32 ± 6	.699
LVEDd, mm	48 ± 6	48 ± 3	49 ± 7	47 ± 6	.754
IVSd, mm	14 ± 3	13 ± 2	14 ± 2	14 ± 5	.784
LPWd, mm	13 ± 3	12 ± 1	13 ± 2	13 ± 4	.48
LVOT diameter, mm	21 ± 2	22 ± 2	21 ± 1	21 ± 2	.624
Prosthesis/LVOT diameter ratio	1.33 ± 0.16	1.21 ± 0.08^**1**^	1.37 ± 0.13^1^, ^2^	1.39 ± 0.20^**2**^	.021
AV area, cm^2^	0.8 ± 0.2	0.9 ± 0.3	0.8 ± 0.2	0.7 ± 0.2	.143
Mean AV gradient, mmHg	42.6 ± 13.0	40.7 ± 8.7	44.3 ± 13.9	42.1 ± 15.7	.853
Peak AV velocity, m/s	4.2 ± 0.6	4.1 ± 0.4	4.3 ± 0.6	4.2 ± 0.8	.694
Pre‐TAVR computed tomography					
Aortic annulus diameter, mm	25.8 ± 1.9	25.2 ± 2.2	26.2 ± 1.8	25.8 ± 1.8	.609
Prosthesis/aortic annulus diameter ratio	1.08 ± 0.09	1.03 ± 0.03	1.08 ± 0.10	1.12 ± 0.09	.109

*Note*: Data presented as mean ± SD, or count (percentage). *P*‐values are based on either Fisher's exact test, one‐way analysis of variance or Kruskal Wallis test. For variables with *P*‐value < .05, only groups with different superscripts are statistically different on post‐hoc testing.

Abbreviations: AV, aortic valve; CABG, coronary artery bypass graft; COPD, chronic obstructive pulmonary disease; IVSd, interventricular septum diameter; LAFB, left anterior fascicular block; LBBB, left bundle branch block; LPFB, left posterior fascicular block; LPWd, left posterior wall diameter; LVEDd, left ventricular end‐diastolic diameter; LVEF, left ventricular ejection fraction; LVESd, left ventricular end‐systolic diameter; LVOT, left ventricular outflow tract; PCI, percutaneous coronary intervention; RBBB, right bundle branch block; TAVR = transcatheter aortic valve replacement; TIA, transient ischemic attack; VP%, ventricular pacing percentage.

**TABLE 2:** TAVR operative details, post‐TAVR electrocardiography and time and indication of permanent pacemaker implantation in patients with low, intermediate, and high percent of ventricular pacing at 6 months

	All patients	VP% <5%	VP% 5%‐95%	VP% >95%	*P*‐value
	(n = 26)	(n = 7)	(n = 10)	(n = 9)	
TAVR operative details					
Valve type: CoreValve Edwards Sapien	6 (23) 20 (77)	0 7 (100)	3 (30) 7 (70)	3 (33) 6 (67)	.257
Prosthesis size 23 mm 26 mm 29 mm 34 mm	2 (8) 11 (42) 10 (39) 3 (12)	2 (29) 3 (43) 2 (29) 0	0 4 (40) 5 (50) 1 (10)	0 4 (44) 3 (33) 2(22)	.501
Preimplantation valvuloplasty	4 (15)	0	2 (20)	2 (22)	.515
Postimplantation dilatation	5 (19)	2 (29)	2 (20)	1 (11)	.828
Pre‐TAVR mean AV gradient	38.7 ± 11.0	44.4 ± 10.1	36.9 ± 12.4	36.2 ± 9.6	.281
Post‐TAVR mean AV gradient	7.6 ± 3.5	7.3 ± 3.0	8.2 ± 3.0	7.1 ± 4.5	.781
Post‐TAVR electrocardiography					
New‐onset LBBB	11 (42)	6 (86)^2^	3 (30)^1^	2 (22)^1^	.039
PR interval, ms	245 ± 61	264 ± 69	197 ± 20	277 ± 61	.271
QRS durations, ms	148 ± 30	166 ± 18	138 ± 39	141 ± 24	.204
Time of PPM implantation					
Postoperative day	4 ± 5	8 ± 8^1^	3 ± 4^1^, ^2^	2 ± 2^2^	.031
Indication of PPM implantation					
Persistent high‐degree AVB	12 (46)	1 (14)^1^	4 (40)^1^	7 (78)^2^	.046
Transient high‐degree AVB	4 (15)	1 (14)	2 (20)	1 (11)	.000
Pause(s) more than 3 s	3 (12)	2 (29)	1 (10)	0	.245
Symptomatic bradycardia	7 (27)	3 (43)	3 (30)	1 (11)	.347

*Note*: Data presented as mean ± SD, or count (percentage). *P*‐values are based on either Fisher's exact test or one‐way analysis of variance. For variables with *P*‐value < .05, only groups with different superscripts are statistically different on post‐hoc testing.

Abbreviations: AV, aortic valve; AVB, atrioventricular block; LBBB, left bundle branch block; PPM, permanent pacemaker; TAVR, transcatheter aortic valve replacement; VP%, ventricular pacing percentage.

**FIGURE 2:**
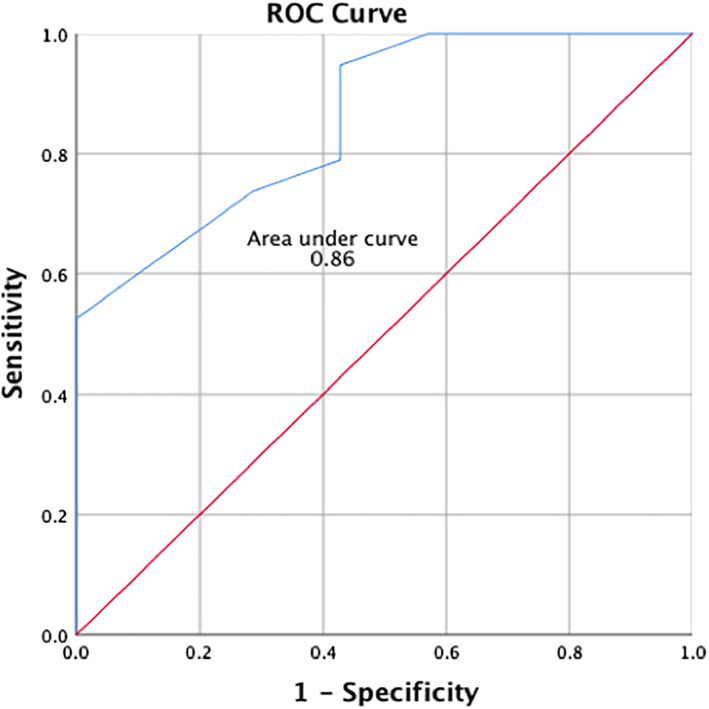
Receiver Operating Curve (ROC) for discriminative ability of TAVR prosthesis/LVOT diameter ratio to predict a percent of ventricular pacing >5% at 6 months after TAVR. LVOT, left ventricular outflow tract; TAVR, transcatheter aortic valve replacement

Using univariate logistic regression analysis (see Table 3), patients who had low VP% <5% over a 6‐months follow‐up period were more likely to have a prosthesis/LVOT diameter ratio < 1.3 (OR 7.00, *P*‐value .048), prosthesis/aortic annulus diameter ratio < 1.02 (OR 7.11, *P*‐value .047), post‐TAVR new‐onset LBBB (OR 16.80, *P*‐value .019), time to PPM implantation greater than 2 days post‐TAVR (OR 9.38, *P*‐value .026), and pre‐TAVR use of a beta blocker (OR 9.40, *P*‐value .026). The cutoff values used for continuous variables were based on ROC curves. An ROC curve was plotted for discriminative ability of prosthesis/LVOT diameter ratio to predict a VP% > 5%, giving an area under curve (AUC) of 0.86 and identifying a highest C‐statistic of 1.3 (Figure 2). Similarly, a value of 1.02 was identified for prosthesis/aortic annulus diameter ratio (AUC 0.74). A multivariable regression analysis model of all significant variables on univariate analysis did not reveal any independent predictors of a low‐VP% <5% at 6 months, likely limited by the small sample size.

**TABLE 3:** Analysis of predictors of low percent of ventricular pacing <5% over a 6 months period after TAVR‐related PPM implantation, using univariate logistic regression analysis

Predictor	Low VP% < 5%	
	OR	*P*‐value
Baseline clinical characteristics		
Age, years	0.96	.445
Body mass index, kg/m^2^	1.10	.191
Hypertension	1.13	.925
Diabetes	1.83	.498
Coronary artery disease	0.62	.593
Heart failure	0.87	.883
Peripheral vascular disease	2.10	.422
COPD	2.81	.276
Serum creatinine	2.29	.435
Beta blocker use	9.40	.026
Calcium channel blocker use	1.12	.908
Pre‐TAVR electrocardiography		
Heart rate, beats/min	0.99	.697
Atrial fibrillation	0.63	.700
PR interval, ms	1.00	.811
QRS durations, ms	0.98	.222
RBBB	0.12	.073
Pre‐TAVR echocardiography		
LVEF, %	1.05	.485
LVESd, mm	0.94	.434
LVEDs, mm	1.01	.916
IVSd, mm	0.87	.483
LPWd, mm	0.71	.216
LVOT diameter, mm	1.28	.329
Prosthesis/LVOT diameter ratio < 1.3	7.00	.048
Mean AV gradient, mmHg	0.98	.647
Peak AV velocity, m/s	0.51	.427
Pre‐TAVR computed tomography		
Aortic annulus diameter, mm	0.79	.363
Prosthesis/Aortic annulus diameter ratio < 1.02	7.11	.047
TAVR operative details		
Prosthesis size: 29 or 34 mm	0.29	.197
Postimplantation dilatation	2.13	.469
Pre‐TAVR mean AV gradient	1.07	.124
Post‐TAVR mean AV gradient	0.97	.792
Post‐TAVR electrocardiography		
New‐onset LBBB	16.80	.019
PR interval, ms	1.01	.376
QRS durations, ms	1.05	.096
Time of PPM implantation		
More than 2 days postoperatively	9.38	.026
Indication of PPM implantation		
Persistent high‐degree AVB	0.12	.073
Transient high‐degree AVB	0.89	.925
Pause(s) more than 3 s	7.20	.136
Symptomatic bradycardia	2.81	.276

Abbreviations: AV, aortic valve; AVB, atrioventricular block; COPD, chronic obstructive pulmonary disease; IVSd, interventricular septum diameter; LBBB, left bundle branch block; LPWd, left posterior wall diameter; LVEDd, left ventricular end‐diastolic diameter; LVEF, left ventricular ejection fraction; LVESd, left ventricular end‐systolic diameter; LVOT, left ventricular outflow tract; PPM, permanent pacemaker; RBBB, right bundle branch block; TAVR, transcatheter aortic valve replacement; VP%, ventricular pacing percentage.

On the other hand, the only predictor of a high‐VP >95% at 6 months was a PPM indication of persistent complete or high‐degree AVB (OR 8.40, *P*‐value .027). In addition, the following variables were not found in any of the patients in the low VP% group at 6 months: preoperative RBBB with bifasicular or trifasicular block, use of self‐expanding prosthetic valve, use of larger 34 mm valve and preimplantation valvuloplasty (see Tables 1 and 2). While these variables are associated with a low odds of having a low‐VP% at 6 months, they were unable to be analyzed by logistic regression.

## 4 DISCUSSION

In this study, we used a single institutional experience to investigate predictors of ventricular pacing burden after TAVR in patients who required PPM implantation. Our findings showed: (i) the incidence of TAVR‐related PPM implantation in our institution was 8.4%, (ii) mean VP% over a 6‐month period was 56‐59%, (iii) a lower median VP% occurred in patients with post‐TAVR new‐onset LBBB at 1, 3 and 6 months, (iv) predictors of low VP% <5% suggestive of PPM independency over a 6‐month follow‐up period included prosthesis/LVOT diameter ratio < 1.3, prosthesis/aortic annulus diameter ratio < 1.02, post‐TAVR new‐onset LBBB, time to PPM implantation greater than 2 days post‐TAVR and pre‐TAVR use of a beta blocker, (v) on multivariable analysis, none of these variables independently predicted a low‐VP%, which may be due to small sample size and model overfitting rather than lack of significance, (vi) on the other hand, a PPM indication of persistent complete or high‐degree AVB predicted a high‐VP% > 95% suggestive of complete PPM dependency over a 6‐month follow‐up period.

The incidence rate of PPM implantation within 30 days after TAVR in our institution (8.4%) is comparable to that reported by the PARTNER 1, 2, and 3 trials that used balloon‐expandable valves (6.6%‐8.8%).^3^, ^5^, ^7^ It is lower however than a lot of recently reported single institutional experiences that used both self‐expanding and balloon expandable valves (incidence rates between 11% and 28%).^10^, ^11^, ^13^, ^14^, ^16^, ^17^ These interinstitutional differences may be partly explained by our higher use of balloon‐expandable valves (77%) which are generally associated with a lower risk of PPM implantation due to their higher level of implantation,^18^ as well as our use of newer generation self‐expanding Evolut valves rather than first generation CoreValves which have a lower risk of PPM implantation.^19^ However despite these factors, there is still a high variability between institutional experiences with rates of PPM implantation after TAVR^19^ which suggests a lack of strong consensus on management of post‐TAVR rhythm disturbances outside the clear indications for PPM implantation. This raises the importance of a better understanding of the natural course of these rhythm disturbances and whether we can identify predictors of pacing requirement with time.

Conduction disturbances after TAVR are believed to be due to mechanical stress from the prosthetic valve on the conduction system in the atrioventricular node and interventricular conduction system.^8^, ^16^ They can be permanent or temporary as a result of postprocedural local inflammation, edema or ischemia that recover with time.^9^ In our institution, 27% of PPM patients had a low VP% <5% at 6 months and there was no significant difference in the mean VP% at 1, 3, and 6 months, which suggests that transient rhythm disturbances probably recover early in the first month postoperatively. Analysis of predictors of high VP% >95% over 6 months (suggestive of complete PPM dependency and a persistent rhythm disturbance) revealed the presence of post‐TAVR persistent complete or high‐degree AV block as a predictor. On the other hand, analysis of predictors of low VP% <5% (suggestive of PPM independency and a transient rhythm disturbance) revealed a number of factors including a lower prosthesis/LVOT diameter ratio or prosthesis/aortic annulus diameter ratio. The association of a higher prosthesis/LVOT diameter ratio with post‐TAVR conduction disturbances has been previously supported in the literature, showing its prediction for new PPM requirement in the PARTNER trial,^7^ and its prediction for PPM dependency after 1 month by Lader et al.^14^ We identified a cutoff value of 1.3 for prosthesis/LVOT diameter ratio and 1.02 for prosthesis/annulus diameter as having most sensitivity and specificity for discrimination, which can be a useful novel tool for clinicians. The difference in values for echocardiography‐derived LVOT diameter and CT‐derived aortic annulus diameter (perimeter‐derived) may be due to lower LVOT measurements due to elliptical aortic annuluses and LV hypertrophy.

Occurrence of post‐TAVR new‐onset LBBB was significantly associated with a low VP% <5% at 6 months in our study. The left bundle branch runs in close proximity to the right coronary leaflet of the AV which makes it susceptible to injury from local inflammation or edema or ischemia from valve deployment.^20^ It occurs in about a third of TAVR patients, yet it is often transient and recovers in over a third of cases before hospital discharge, probably due to resolution of periprocedural inflammation.^21^ The association of a new‐onset LBBB with a lower VP% over 6 months suggests that the associated rhythm disturbances are transient and recover in the early postoperative period. This is a finding that was supported by Lader et al. who found that having a post‐TAVR LBBB with subsequent development of complete AVB was associated with return of normal conduction within 1 month.^14^ These findings support the notion that the presence of a post‐TAVR LBBB should not be necessarily be alone interpreted as an indicator for PPM implantation.

Similarly to post‐TAVR new‐onset LBBB, later time to PPM implantation greater than the median time of 2 days post‐TAVR was significantly associated with a low‐VP% <5% at 6 months. A similar association of earlier time to PPM implantation predicting PPM dependency was reported by Lader et al.^14^ as well as other single‐center studies.^10^, ^13^ Variables that showed a trend toward predicting a high‐VP% at 6 months in our institution included a PPM indication of persistent complete or high‐degree AVB, preoperative RBBB with bifasicular or trifasicular block, use of a self‐expanding prosthetic valve, use of a large 34 mm valve and preimplantation valvuloplasty. The significance of pre‐TAVR RBBB is especially important, as it is the most persistently reported predictor of PPM requirement in the literature,^7^, ^9^, ^17^ as well as often being reported as a predictor of PPM dependency.^10^, ^11^, ^14^

### 4.1 Limitations

While our study raises some interesting findings, several limitations need to be considered to place these findings in the proper perspective. The main limitation lies in our small sample size which influences the statistical power of our analysis especially for multivariable analysis and the ROC curve. The retrospective design of the study makes us unable to account for unidentified or unmeasured confounding variables as depth of valve implantation, and our single institutional experience with its predominantly male population weakens the ability to generalize our findings. On the other hand, single institutional experiences in this field are particularly valuable to allow us to investigate deeper into the reasons for variability in TAVR PPM rates between different institutions. Last, while percentage of ventricular pacing may not be an accurate measure of PPM dependency compared to assessment of the presence of intrinsic ventricular activity, it provides a good assessment of pacing requirement over time.

## 5 CONCLUSIONS

In this small sample single‐institution study, a lower TAVR prosthesis/LVOT or aortic annulus diameter ratio, post‐TAVR new‐onset LBBB and later time to PPM requirement showed a trend toward predicting a low‐VP% at 6 months when a PPM was indicated. This observation may suggest prediction for PPM independency, and may help in the clinical decision‐making process when a PPM is being considered for TAVR‐related conduction disturbances where a strong consensus is not available.

## CONFLICTS OF INTEREST

The authors declare no potential conflict of interests.

## Supporting information


**Supplementary Figure 1** Measurement of aortic annulus diameter (derived from perimeter) on computed tomography, compared to measurement of left ventricular outflow tract diameter on transthoracic echocardiography
