# Supplementary figures and images for: Predictors of ventricular pacing burden after permanent pacemaker implantation following transcatheter aortic valve replacement
Source: Clin Cardiol. 2020 Sep 4;43(11):1334–42. doi: 10.1002/clc.23447 (PMC7661645; doi:10.1002/clc.23447)

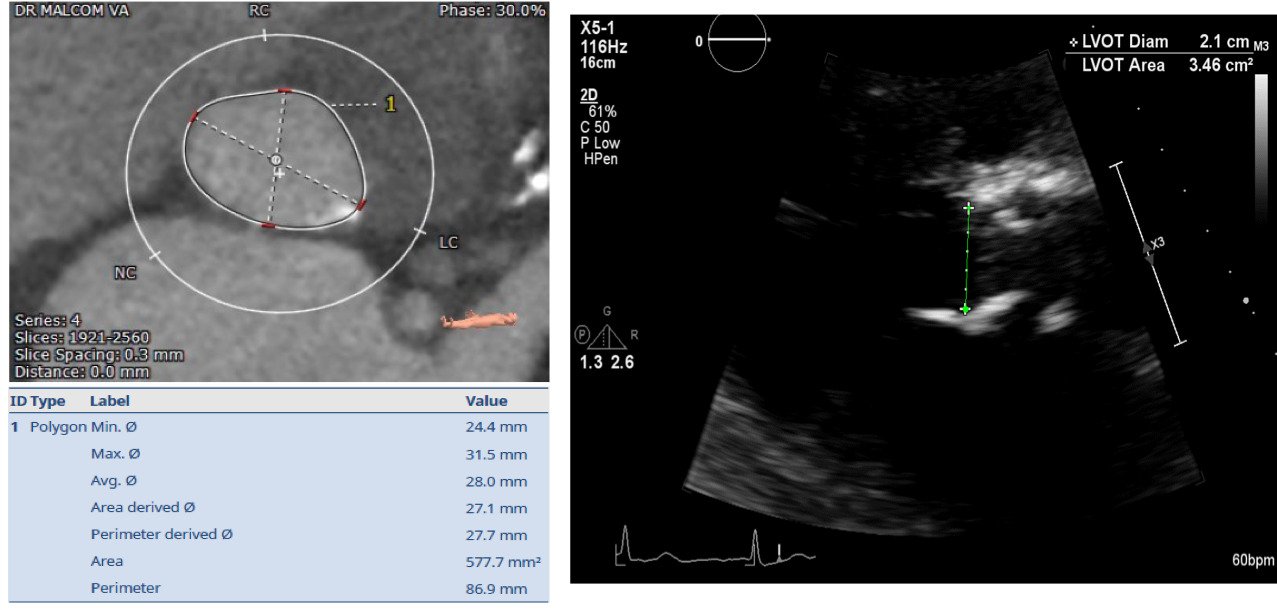

Supplement: Supplementary file 1 — Supplementary Figure 1 Measurement of aortic annulus diameter (derived from perimeter) on computed tomography, compared to measurement of left ventricular outflow tract diameter on transthoracic echocardiography [file CLC-43-1334-s001.jpg]
